# Probiotic *Hungatella hathewayi* increases host estrogen level via regulation of gut microbiota and host metabolism in sows

**DOI:** 10.3389/fmicb.2025.1598365

**Published:** 2025-06-06

**Authors:** Kai Shi, Xiao Zhou, Jiuli Dai, Jiawei Jia, Guozeng Dong, Yongheng Wang, Yangyang Shen, Shufang Chen

**Affiliations:** ^1^Ningbo Academy of Agricultural Sciences, Institute of Livestock and Poultry Research, Ningbo, China; ^2^Ningbo Key Laboratory of White Goose Germplasm Resource Innovation and Ecological Farming, Ningbo, China; ^3^Xiangshan Anji Animal Husbandry and Veterinary Service Co., LTD, Ningbo, China; ^4^Ningbo GooseBeller Poultry Industry Technology Development Co., LTD, Ningbo, China; ^5^Institute of Animal Science, Jiangsu Academy of Agricultural Sciences, Nanjing, China

**Keywords:** *Hungatella hathewayi*, sows, reproduction, hormones, multi-omics

## Abstract

**Introduction:**

Animal reproduction is a highly complex process influenced by numerous factors, and previous study has suggested that *Hungatella hathewayi* (HH) may have potential benefits for swine reproduction. Building on these findings, the objective of this study was to investigate the effects of HH supplementation on sows’ hormone levels, antioxidant capacity, host metabolism, and gut microbiota.

**Methods:**

Twenty healthy Large-Yorkshire sows with similar physical conditions were randomly divided into four groups based on the principle of similar weight (*n = 5*). The control group was fed a basal diet, while the treatment groups received the basal diet supplemented with 5 × 10^10^, 5 × 10^11^, and 5 × 10^12^ CFU/sow of HH. Supplementation with HH was administered every three days over a treatment duration of 30 days. Serum and feces of sows were collected at the end of the experiment.

**Results:**

Dietary HH supplementation significantly increased the estrogen concentration in sows but did not alter the levels of FSH, progestogen, or antioxidative capacity (T-AOC, SOD, and MDA). 16S rRNA sequencing indicated that HH treatment altered the gut microbial composition and metabolism, increasing the relative abundance of Roseburia, *Alloprevotella*, *Lachnospira*, *Anaerovibrio*, and *Hungatella* in the HH group. Further metabolomic analysis suggested that the differentially accumulated metabolites from serum and feces involved changes in the metabolism of pyrimidine and tryptophan, as well as alterations in steroid hormone biosynthesis.

**Discussion:**

Our findings suggest that dietary supplementation with *Hungatella hathewayi* has the potential to modulate host estrogen levels through the regulation of gut microbiota and host metabolism. This mechanism may serve as a novel and promising approach for influencing reproductive performance in sows.

## 1 Introduction

Reproduction is essential for the continuity and survival of populations, representing a complex process that is influenced by a multitude of factors, including endocrine hormones (Leng et al., 2024), genetics (Rodrigues et al., 2020), nutritional status (Castillo-Matamoros and Poveda, 2021), and others. Hormones derived from the hypothalamic-pituitary-gonadal (HPG) axis are pivotal in regulating both humans and animals’ follicular development, oocyte maturation, and ovulation (Plant, 2015). Gonadotropin-releasing hormone (GnRH), secreted by the hypothalamus, exerts its effects on gonadotrophin cells in the anterior pituitary gland, leading to the synthesis and secretion of luteinizing hormone (LH) and follicle-stimulating hormone (FSH), which collectively promote estrogen production within ovarian follicles (Mikhael et al., 2019). Additionally, estrogen acts on specific neuronal populations in the hypothalamus to modulate the release of GnRH and the subsequent secretion of FSH and LH (Ae, 2020). Beyond the HPG hormones, a diverse array of peptides exerts significant roles in regulating animal reproduction. For example, prolactin (PRL), a pituitary-derived hormone, can inhibit luteinizing hormone (LH) secretion (Cohen-Becker et al., 1986; Ladyman et al., 2020), while neuropeptide Y (NPY), a key neuropeptide in the central nervous system, stimulates the release of gonadotropin-releasing hormone (GnRH) and LH through central neural pathways (Evans et al., 2001).

The gastrointestinal tract harbors a diverse array of microbiota, and numerous large-scale studies have demonstrated that gut microbiota significantly influence fertility through influencing host metabolism (Shi et al., 2024b). Comparative analyses of fecal microbiota from sows with differing reproductive productivity have revealed variations in short-chain fatty acids (SCFAs)-producing and fiber-degrading bacteria, such as *Ruminococcus*, *Fibrobacter*, and *Butyricicoccus*. Furthermore, higher concentrations of SCFAs were observed in sows characterized by greater fecundity (Uryu et al., 2020). Gut microbiota can utilize host intestinal food residues to synthesize neurotransmitters, SCFAs, gut hormones, and branched-chain amino acids, influencing the host’s endocrine, nervous, and immune systems (Loh et al., 2024). Consequently, the gut microbiota may mediate the beneficial effects of inulin and cellulose supplementation in host diets, thereby promoting ovarian oocyte maturation (Men et al., 2022). Additionally, studies involving fecal microbiota transplantation from younger to older animals have indicated that the older subjects exhibited enhanced fertility and decreased follicular atresia and apoptosis. At the same time, their offspring displayed improved motor abilities (Hu et al., 2022; Xu et al., 2022). Despite these findings, there are relatively few studies exploring the effects of specific microbes on female reproductive health. In our previous research, we observed an increase in *Hungatella* levels during the peak-laying period in hens, which was positively correlated with estrogen concentrations in host cecal contents (Shi et al., 2024a). Moreover, the abundance of *Hungatella* was significantly elevated in females with endometriosis, who exhibited higher levels of estrogen metabolites, implying a potential relationship between *Hungatella* and host estrogen (Pai et al., 2023). Another investigation indicated that *Hungatella hathewayi* may facilitate the return of estrus in sows by regulating estrogen levels (Liu et al., 2023). *Hungatella hathewayi*, a species within the *Hungatella* genus, harbors beta-glucuronidase, which could potentially influence circulating estrogen levels, although further research is needed to confirm this relationship.

The pig exhibits physiological reproductive events that closely parallel those of multi mammalians, making it an excellent model for investigating the mechanisms of reproduction (Mordhorst and Prather, 2017). Consequently, this study aimed to explore the potential effects of *Hungatella hathewayi* on sows’ hormonal levels and antioxidative capacity. Furthermore, we aimed to detect changes in fecal microbial composition and differences in the metabolomic profiles of feces and serum to elucidate the regulatory mechanisms of *Hungatella hathewayi* on reproduction.

## 2 Materials and methods

### 2.1 Animals and treatment

All procedures in this experiment were approved by the Animal Care and Use Committee of the Ningbo Academy of Agricultural Sciences (approval number NKYLL-2024-02). Twenty healthy Large White × Yorkshire sows, aged 9 months, were randomly selected and assigned to four groups following the principle of similar weight. The experiment spanned one month. The four study groups were as follows: (1) sows fed with a basal diet (Control); (2) sows fed with a basal diet supplemented with HH [5 × 10^10^ colony-forming units (CFU) per sow in sterile PBS]; (3) sows fed with a basal diet supplemented with 5 × 10^11^ CFU HH; (4) sows fed with a basal diet supplemented with 5 × 10^12^ CFU HH. The concentrations of HH used in this study were based on previous studies (Reagan-Shaw et al., 2008; Li et al., 2020). *Hungatella hathewayi* (HZBMC, HZbio, Microbial Conservation; catalog number HZB432244) was obtained and cultured anaerobically in PYG medium (104b, Leibniz-Institut DSMZ GmbH, Braunschweig, Germany) at 37°C for 12–14 h. All sows had *ad libitum* access to feed and water. The nutritional composition of the sows’ diet is detailed in Table 1.

**TABLE 1:** The nutritional composition of the sows’ diets.

Nutrition composition	Percentage (%)
Corn gain	57
Secondary wheat powder	10
Bran	8
Soybean meal	18
Fish meal	3
Trace minerals	1
CaHPO_4_	0.7
Zeolite powder	2
Salt	0.3

### 2.2 Sample collection

Following a one-month trial, blood samples were collected and allowed to stand at room temperature for 30 min to facilitate clotting. Subsequently, the serum was separated by centrifugation at 1,500 × *g* for 5 min at 4°C. Fecal samples were also collected from the sows after the trial. All samples were stored at −80 °C for subsequent analysis.

### 2.3 Hormone and antioxidative capacity detection

Serum samples were utilized to determine the concentrations of progestogen, follicle-stimulating hormone (FSH), and estrogen, as well as the levels of total antioxidant capacity (T-AOC), glutathione (GSH), and malondialdehyde (MDA). These analyses were performed using commercial assay kits provided by Shanghai Enzyme-linked Biotechnology Co., Ltd. Absorbance measurements of the serum samples were conducted using a microplate reader (Tecan, Männedorf, Switzerland).

### 2.4 Microbial DNA extraction and 16S rRNA sequencing

The genomic DNA of sows’ feces was extracted using the MolPure^®^ Stool DNA Kit (YEASEN Biotech, Shanghai, China) following the manufacturer’s guidelines, extracted DNA was quantified using a NanoDrop2000 spectrophotometer (Thermo Fisher Scientific, DE, USA) and qualified using 1% agarose gel electrophoresis.

The V4 hypervariable regions of the microbiota 16S rRNA gene were amplified using specific primers 515F (GTGCCAGCMGCCGCGGTAA) and 806R (GGACTACHVGGGTWTCTAAT). All PCR reactions were conducted using 15 μL of Phusion High-Fidelity PCR Master Mix from New England Biolabs, with 0.2 μM of both forward and reverse primers and approximately 10 ng of template DNA. The thermal cycling protocol commenced with an initial denaturation step at 98°C for 1 min, succeeded by 30 cycles consisting of denaturation at 98°C for 10 s, annealing at 50°C for 30 s, and extension at 72°C for 30 s. A final extension step was performed at 72°C for 5 min. PCR products were purified using a Qiagen Gel Extraction Kit manufactured in Germany. Sequencing libraries were prepared employing the TruSeq DNA PCR-Free Sample Preparation Kit from Illumina, USA, adhering to the supplier’s guidelines, with index codes appended. The quality of the libraries was evaluated using a Qubit 2.0 Fluorometer from Thermo Scientific and an Agilent Bioanalyzer 2100 system. Ultimately, the libraries were sequenced on an Illumina NovaSeq platform, yielding 250-bp paired-end reads.

### 2.5 16S rRNA data analysis

In this study, the processing of primer sequences was conducted using Vsearch (version 2.14.1) and Usearch (version 11.0.667), which are widely recognized tools for handling high-throughput sequencing data. The raw sequencing data underwent a series of steps, including trimming to remove primer sequences, filtering to eliminate low-quality sequences, dereplication to remove duplicate sequences, and denoising to correct sequencing errors, resulting in high-quality clean amplicons. These clean amplicons were then used for taxonomic annotation against the SILVA database (release 132_99), a comprehensive and authoritative database for microbial classification.

The diversity of microbial communities was evaluated through the computation of alpha and beta diversities using Usearch. Alpha diversity, which reflects the richness and evenness of microbial communities, was assessed using Shannon and richness indices. Beta diversity, which indicates the variation among different microbial communities, was analyzed using constrained principal coordinates analysis (cPcoA) to compare intestinal microbiota across different experimental groups.

To identify differentially abundant microbes between groups, STAMP (version 2.1.3) software was employed for statistical analysis. Additionally, linear discriminant analysis (LDA) coupled with the LEfSe tool was used to detect microbial biomarkers that significantly differed across groups. The LEfSe tool, accessible at https://www.bic.ac.cn/BIC/#/analysis?page=b%27MzY%3D%27&tool_type=tool (accessed on 22 October 2024), was utilized for this purpose. Finally, the phylogenetic investigation of Communities Reconstruction of Unobserved States (PICRUSt2) was employed to predict the functional potential of the fecal microbiota based on 16S rRNA gene sequences (Douglas et al., 2020).

### 2.6 Metabolomic sequencing and data analysis

Serum samples (100 μL) were aliquoted into EP tubes and resuspended with prechilled 80% methanol using vigorous vortexing. Following this, the samples were placed on ice for 5 min to allow for complete precipitation, then centrifuged at 15,000 *g* at 4°C for 20 min. A portion of the supernatant was diluted to a final concentration containing 53% methanol using LC-MS grade water. The samples were transferred to new Eppendorf tubes and centrifuged again at 15,000 *g* at 4°C for 20 min. The supernatant was then carefully injected into the LC-MS/MS system for positive and negative ion modes analysis to facilitate relative quantification. Metabolites were identified and quantified by comparing their retention times and mass-to-charge ratios (m/z) with those in reference databases. The metabolomic data were processed by subtracting background ions as determined by blank samples and normalizing the data. The identified metabolites were annotated using the KEGG,^1^ HMDB,^2^ and LIPIDMaps^3^ databases. Metabolites were deemed differential if they exhibited a Variable Importance in Projection (VIP) score exceeding 1, a *P*-value below 0.05, and a fold change of at least 2 or no more than 0.5. The roles of these metabolites were then further elucidated using the MetaboAnalyst online platform (version 6.0).

### 2.7 Statistical analysis

The hormone data of sows were processed and analyzed using one-way analysis of variance (ANOVA) with the SPSS 23.0 software package (IBM, Armonk, NY, USA). The results were visualized as the mean ± standard deviation (SD) using Prism (v.8.0; GraphPad Software, San Diego, CA, USA). Statistical significance was determined using a *P*-value threshold of less than 0.05.

## 3 Results

### 3.1 Effect of *Hungatella hathewayi* on host hormones levels and antioxidative capacity

**FIGURE 1:**
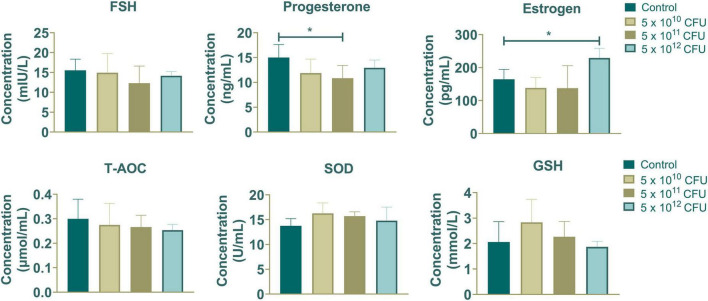
The contents of hormones (FSH, progesterone and estrogen) and antioxidative capacity (T-AOC, SOD and GSH) in sows supplemented with different concentrations of *Hungatella hathewayi*. * indicated that the *P*-value < 0.05.

As depicted in Figure 1, dietary supplementation with *Hungatella hathewayi* did not significantly alter the concentration of FSH. However, it prominently decreased the progesterone level at a dosage of 5 × 10^11^ CFU and increased the concentration of estrogen at 5 × 10^12^ CFU (*P*-value < 0.05). The levels of T-AOC, SOD and GSH indicated that supplementation with *Hungatella hathewayi* did not significantly change the host’s antioxidative capacity. Subsequently, serum and fecal samples from both the control and treatment groups that received a dose of 5 × 10^12^ CFU/sow of HH were subjected to further analysis.

### 3.2 Difference in sows’ fecal microbiomes after dietary *Hungatella hathewayi* supplementation

**FIGURE 2:**
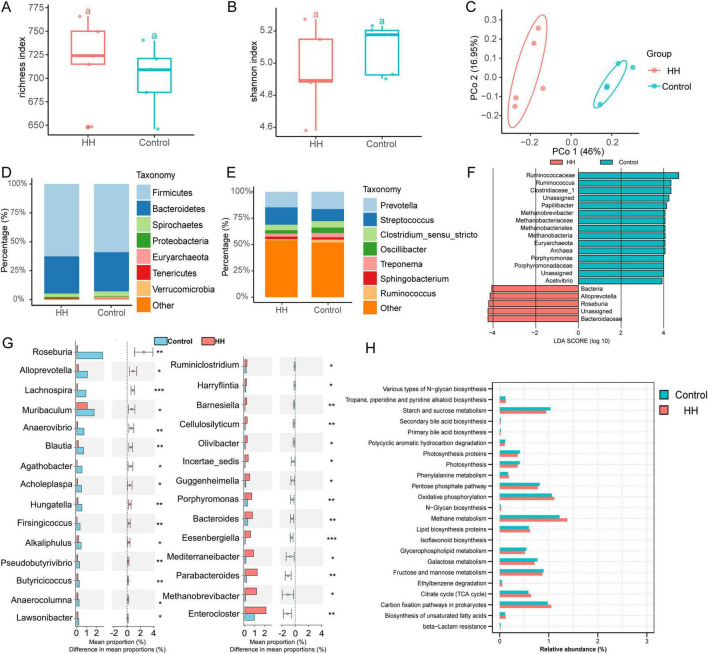
The effect of HH supplementation on sows’ feces microbiota. Richness **(A)**, Shannon index **(B)** and Principal coordinates analysis **(C)** of fecal microbes. The feces microbial composition at the phylum **(D)** and genus **(E)** levels. Identification of microbial biomarkers **(F)** and differential fecal microbiota **(G)** between the control and HH groups. KEGG pathway analysis of 16S rRNA sequencing in the two groups **(H)**. *P*-value < 0.05 suggested a significant difference. * represented *P*-value < 0.05; ** suggested *P*-value < 0.01; *** indicated that the *P*-value < 0.001.

16S rRNA sequencing indicated that the microbial richness index increased, while the Shannon diversity index decreased in the HH group (*P*-value > 0.05) (Figures 2A, B). Microbial beta diversity analysis suggested alterations in fecal microbial composition (Figure 2C). At the phylum level, Firmicutes, Bacteroidetes, Spirochaetes, and Proteobacteria were the dominant phyla in both the control and HH groups (Figure 2D). At the genus level, *Prevotella*, *Streptococcus*, *Clostridium_sensu_stricto*, and *Oscillospira* were highly abundant in both groups (Figure 2E).

Linear discriminant analysis effect size (LEfSe) analysis identified the family Ruminococcaceae as a biomarker in the control group, and the family Bacteroidaceae was recognized as a key bacterium in the HH group (Figure 2F). A total of 29 genera differed significantly between the control and HH groups, with the relative abundances of *Roseburia*, *Alloprevotella*, *Lachnospira*, *Anaerovibrio*, and *Hungatella* being significantly increased following dietary supplementation with *Hungatella hathewayi* (Figure 2G). Additionally, 23 KEGG pathways, including those involved in methane and starch and sucrose metabolism, were significantly altered between groups (Figure 2H).

### 3.3 *Hungatella hathewayi* changed the sow’s fecal metabolic profile

**FIGURE 3:**
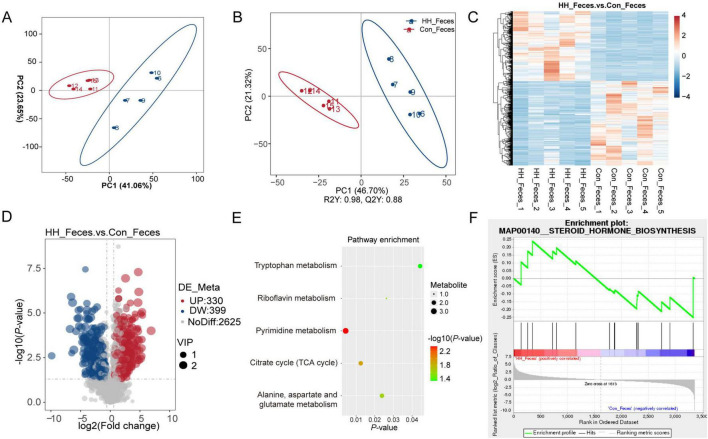
Metabolome analysis of sows’ feces after HH supplementation. PCA **(A)** and PLS-DA **(B)** plots of metabolomic assay of the control and HH groups. Heatmap of hierarchical clustering to differentiated metabolites in the two groups **(C)**. Volcano plot **(D)** and KEGG pathway **(E)** of differential metabolites between the control and treatment groups. Metabolite-Enriched Gene Set Enrichment Analysis (MetGSEA) displayed the changed pathway in different groups **(F)**.

In our metabolomic sequencing results, a total of 3,354 metabolites were identified. Principal component analysis (PCA) and partial least squares discriminant analysis (PLS-DA) indicated significant differentiation in the fecal metabolite profiles between the control and HH groups (Figures 3A, B). A total of 729 differentially abundant metabolites (DAMs) were identified through metabolomics analysis, 330 DAMs were significantly upregulated in the HH group, while 399 DAMs were downregulated in the control group (Figures 3C, D). KEGG pathway analysis revealed that these differential metabolites were associated with several key metabolic pathways, including pyrimidine metabolism, tryptophan metabolism, and riboflavin metabolism (Figure 3E). Furthermore, Gene Set Enrichment Analysis (GSEA) suggested that the steroid hormone biosynthesis pathway was altered between the control and HH groups (Figure 3F).

### 3.4 The effect of *Hungatella hathewayi* on host serum metabolome

**FIGURE 4:**
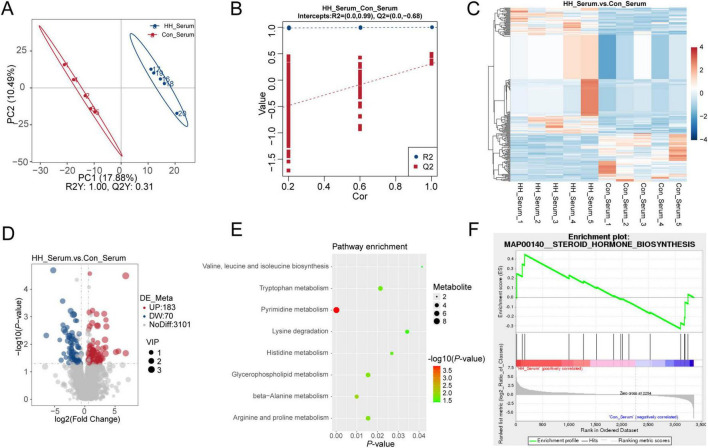
Metabolome analysis of sows’ serum in the control and HH groups. PLS-DA between the two groups **(A,B)**. Hierarchical cluster analysis **(C)** and volcano plot of DAMs **(D)**. KEGG pathway **(E)** and MetGSEA **(F)** of differential metabolites between the control and HH groups.

The PLS-DA model demonstrated that serum metabolites in the control and HH groups had distinct profiles in both positive and negative ion modes (Figures 4A, B). Comparative analysis revealed 183 upregulated and 70 downregulated differentially accumulated metabolites (DAMs) in the HH group relative to the control group (Figures 4C, D). These DAMs were associated with metabolic pathways such as pyrimidine metabolism, tryptophan metabolism, histidine metabolism, and beta-Alanine metabolism (Figure 4E). Furthermore, GSEA analysis indicated that the steroid hormone biosynthesis pathway was significantly altered in serum between the control and HH groups (Figure 4F).

## 4 Discussion

Reproduction is vital for genetic transmission and species survival, estrogen secreted from HPG axis could promote the development of reproductive organs and maintaining secondary sexual characteristics (Kaiser et al., 1997). However, an increasing number of studies found that microbiota interact with the host to regulate circulating estrogen levels (Hu et al., 2023; Wang et al., 2024). Circulating estrogen undergoes metabolic inactivation in the liver and is subsequently excreted through the gut, while gut microbiota containing β-glucuronidase (gmGUS) can re-activate estrogen and influence host estrogen levels (Ervin et al., 2019). Gut microbiota dysbiosis reduced microbial diversity can lead to decreased levels of gmGUS, further altering estrogen levels (Qi et al., 2021). The intestinal microbiota community is also affected by the circulating estrogen levels (Kumari et al., 2024); it has been reported that the abundances of Gammaproteobacteria and *Myxococcales* are positively correlated with estrogen, while *Prevotellaceae* show a negative relationship with estrogen (Santos-Marcos et al., 2018). *Hungatella hathewayi* contains β-glucuronidase, and its abundance has been found to correlate with host estrogen levels in sows (Liu et al., 2023). In this study, we demonstrated that the supplementation of *Hungatella hathewayi* at a dosage of 5 × 10^12^ CFU/sow significantly increased estrogen levels in sows. However, the levels of T-AOC, SOD, and GSH, which are key indicators of host antioxidant capacity, remained unchanged following HH supplementation. This suggests that while HH may influence estrogen metabolism, it does not significantly affect the host’s antioxidant status. Previous studies have suggested that gavage with *Hungatella hathewayi* could alter serum taurine levels and protect the host from intracranial aneurysm formation (Li et al., 2020). Our findings suggest that *Hungatella hathewayi* has the potential to serve as a safe additive for modulating host circulating estrogen levels.

Dietary supplementation with *Hungatella hathewayi* significantly altered the gut microbiota composition, with notable increases in the relative abundances of *Roseburia*, *Alloprevotella*, *Lachnospira*, and *Hungatella* in the HH group, leading to functional changes in methane metabolism, starch metabolism, and sucrose metabolism. *Roseburia*, a key intestinal microbe known for its production of SCFAs, influences host metabolism and has been observed to decrease during pregnancy (Koren et al., 2012; Kang et al., 2023). *Alloprevotella*, another SCFAs-producing bacterium, has been implicated in affecting female cervical ripeness during pregnancy (Lu et al., 2024), and its abundance has been found to significantly differ between primiparous and multiparous sows (Gaukroger et al., 2021). Furthermore, *Alloprevotella* and *Anaerovibrio* were abundant in the gut during host pregnancy, and the pathways involved in the production of the short-chain fatty acid butyrate were increased (Rhoades et al., 2022). *Lachnospira*, a key microbe influencing nutrient intake and body weight in pregnant women, is particularly relevant to fetal delivery and growth (Stanislawski et al., 2017; Kunasegaran et al., 2023). Our findings indicate that supplementation with *Hungatella hathewayi* appears to influence host circulating estrogen levels through interactions with other gut microbiota.

Metabolomic analysis identified 253 and 729 DAMs in serum and feces, respectively, which are known to regulate pyrimidine and tryptophan metabolism. Additionally, the steroid hormone biosynthesis pathway was altered in both serum and feces. Previous research has indicated that changes in pyrimidine and tryptophan metabolism are observed in follicular fluid, serum, and urine of sows with varying reproductive performance, suggesting that these metabolic pathways may play a role in regulating reproductive outcomes in sows (Chen et al., 2019). In addition, several intermediate metabolites of pyrimidine metabolism have been established as regulators of host reproductive signaling (Wan et al., 2019). In perimenopausal women, alterations in gut microbiota have been associated with changes in concentrations of FSH, LH, estrogen, and progesterone, as well as altered fecal amino acid metabolism (tyrosine, tryptophan) and lipid metabolism (alpha-linolenic acid metabolism) (Xie et al., 2024). Another study found that tryptophan derivatives decreased host estradiol and FSH synthesis, impeding ovulation and corpus luteum formation, ultimately disrupting maternal reproductive performance (Shen et al., 2023). Furthermore, tryptophan supplementation has improved sows’ average daily feed intake, milk yield, and reproductive performance (Miao et al., 2019). Collectively, these results suggest that dietary supplementation with *Hungatella hathewayi* could be a potential additive to influence host hormone levels and, consequently, reproductive performance.

## 5 Conclusion

This study demonstrated that dietary supplementation with *Hungatella hathewayi* significantly increased the estrogen levels in sows and did not disrupt the host’s antioxidative capacity. 16S rRNA sequencing revealed that *Hungatella hathewayi* supplementation influenced the gut microbial composition and altered metabolic pathways. Subsequent metabolomic analyses suggested that host pyrimidine, tryptophan metabolism, and steroid hormone biosynthesis were affected. These findings present *Hungatella hathewayi* as a potential dietary supplement to regulate host reproductive performance.

## Data Availability

The 16S rRNA sequencing data utilized in this study can be accessed at the China National Center for Bioinformation (https://www.cncb.ac.cn/), and are archived under the Genome Sequence Archive (GSA) with the accession number CRA021453. The Metabolomics data can be accessed via Figshare under the following DOI https://doi.org/10.6084/m9.figshare.29107922.v1.

## Footnotes

1
https://www.genome.jp/kegg/pathway.html

2
https://hmdb.ca/metabolites

3
http://www.lipidmaps.org/
